# Comparison of the self-administered and interviewer-administered modes of the child-OIDP

**DOI:** 10.1186/1477-7525-6-40

**Published:** 2008-06-02

**Authors:** Georgios Tsakos, Eduardo Bernabé, Kevin O'Brien, Aubrey Sheiham, Cesar de Oliveira

**Affiliations:** 1 Department of Epidemiology and Public Health, University College London, London, UK; 2 Departamento de Odontología Social, Universidad Peruana Cayetano Heredia, Lima, Peru; 3 Dental School, University of Manchester, Manchester, UK

## Abstract

**Background:**

The mode of questionnaire administration may affect the estimates and applicability of oral health-related quality of life indicators. The aim of this study was to compare psychometrically the self-administered Child-OIDP index with the original interviewer-administered instrument.

**Methods:**

This was a cross-sectional study of 144 consecutive children aged 9–16 years referred to orthodontic clinics in Bedfordshire. To compare the two administration modes of the Child-OIDP, the sample was randomly split in two groups. The two groups were analysed in terms of baseline characteristics, self-perceived measures (self-rated oral health, self-perceived need for braces, happiness with dental appearance, frequency of thinking about dental appearance), Child-OIDP performance scores and overall score and psychometric properties (criterion validity and internal reliability).

**Results:**

No significant difference between the two groups was found in relation to their sociodemographic profile and self-perceived measures. The self- and interviewer-administered Child-OIDP had identical mean scores and did not differ in recording any of the eight performances (p ≥ 0.206). For criterion validity, the correlation coefficients of the Child-OIDP with self-perceived measures were not different between the two modes of administration (p ≥ 0.118). Furthermore, the Cronbach's alpha values of the two groups were similar (p = 0.466).

**Conclusion:**

This study demonstrated that the self-administered Child-OIDP performed the same as the original interviewer-administered mode, while at the same time reducing administration burden. This provides support for the use of the self-administered Child-OIDP. Further studies should focus on a more comprehensive psychometric evaluation.

## Background

This study assesses differences between two different administration modes of an oral health-related quality of life (OHRQoL) measure for children. The Child-OIDP [1] is an interviewer-administered OHRQoL measure that assesses the frequency and severity of oral impacts on eight daily life performances. Through its condition-specific feature, where the oral impacts are attributed to specific oral conditions according to the respondent's perceptions, the Child-OIDP can be used in needs assessment and for planning services [2]. Indeed, its usefulness has been demonstrated in assessing general paedodontic treatment needs [3], as well as orthodontic treatment needs [4].

The effect of the mode of questionnaire administration on the estimates of health-related indicators is important [5,6]. In general, interviewer-administered questionnaires are associated with higher response rates compared to self-administered instruments. On the other hand, they are also characterised by higher administration costs, hence limiting their practical applicability. Furthermore, interaction between respondent and interviewer may introduce bias in the estimates, while self-administered questionnaires may suffer from respondent bias, through for example exclusion of participants with reading difficulties. However, the most important conceptual issue relates to the comparability between data collected with interviewer- and self-administered questionnaires [5-7].

Studies indicate that in general self-administered questionnaires and face-to-face interviews provide similar estimates of self-assessed status [7-10], but others have expressed a preference for face-to-face interviews [11]. Previous studies comparing self- and interviewer-administered modes of health-related quality of life questionnaires showed that both performed successfully in terms of psychometric properties [12,13]. However, studies have also shown differences between interviews and self-administrations, with the former providing a more favourable picture of quality of life [6,12-15]. A study on OHRQoL that used interviews and self-administrations of two measures (the OIDP and the OHIP-14) in a primary care department of a dental hospital showed that OIDP overall scores were unrelated to the administration mode [16]; however, no comparison of psychometric properties was carried out.

In child populations in particular, the assessment of OHRQoL should be considered in the light of the cognitive development of children [17], especially as complex language or conditional sentences do not become common until the age of 11–12 years [18]. Therefore, child-specific measures should avoid using complex constructs if they are to be applied to younger age groups. Indeed, quality of life measures in relation to both general and oral health have performed satisfactorily as self-administered instruments in even younger populations [19,20]. The administration of the Child-OIDP involves an individual face-to-face interview with each child. In order to reduce interview time and respondent burden, the use of pictures have been used in the Thai version of Child-OIDP [1,21] while a shorter version of the OIDP based only on the assessment of frequency of oral impacts has been used in other settings [22,23]. A self-administered Child-OIDP questionnaire would be more practical and cost effective than the current face-to-face interview and would further facilitate the applicability of the instrument in both clinical practice and population epidemiological survey settings. Therefore, the aim of this study was to compare psychometrically the self-administered Child-OIDP with the original interviewer-administered instrument.

## Methods

### Sample design

This was a cross-sectional study of 144 consecutive children aged 9–16 years referred to orthodontic clinics in the Bedfordshire Personal Dental Service (PDS) for orthodontic diagnosis and treatment. By consecutive, we mean that all children referred to orthodontic clinics were included in the study. The Orthodontic Personal Dental Services (PDS) Pilot Scheme in Bedfordshire Heartlands Primary Care Trust (PCT) involves independent orthodontic practitioners with a contract with the local PCT. Its aim was to prioritise and provide orthodontic services to children with the greatest oral health needs [24]. The study focussed on a sample of orthodontic patients, as this is an important oral health issue at this age group. Furthermore, a patient rather than a school-based sample would be expected to have higher prevalence of oral impacts and would also allow for the assessment of practicality of using the self-administered Child-OIDP in a clinical setting.

Ethical approval was obtained from the Brent Medical Ethics Committee, the Research and Development Panel of the Bedfordshire Heartlands Primary Care Trust and the Research and Development Directorate of the University College London Hospitals National Health Service Trust.

For practical reasons, both versions of the Child-OIDP questionnaire were administered on the same visit and with the same order of administration (self-administered first, interviewer-administered second). This may introduce bias in the responses of the participants and result in extensive agreement between the two versions. Consequently, in order to address this potential bias and assess whether there are differences between the two modes of the Child-OIDP administration, namely the self- versus the interviewer-administered questionnaire, the sample was randomly split into two groups of 72 children each and one Child-OIDP version was used per child. The self-administered version from one group, hereafter named the Self-administered Questionnaire (SAQ) group was compared with the interviewer-administered version from the other group, hereafter named Face-To-Face Interview (FTFI) group.

### Data collection

For the Child-OIDP interview [1,25], children were first given a list of common oral problems and were asked whether they had experienced any of them within the last 3 months. Then, the single interviewer asked about difficulties in daily life caused by the problems that they marked on the list. The impacts of oral problems on daily life were quantified by using frequency and severity scores (scales from 1 to 3) for difficulty in carrying out 8 daily life performances (eating, speaking, cleaning mouth, sleeping, emotion, smiling, study, and social contact). If no impact was reported, then a zero score was assigned. Performance scores were calculated by multiplying the frequency and severity scores, while the overall Child-OIDP score is the sum of the 8 performance scores (ranging from 0 to 72) multiplied by 100 and divided by 72. To facilitate its appropriateness for self-completion, the self-administered Child-OIDP used a different, more user-friendly layout, with clear guiding instructions throughout the questionnaire, than the interviewer-administered Child-OIDP. In addition, the content and language were slightly simplified by avoiding some technical terms (e.g. in the common oral problems list, "erupting permanent tooth" was changed into "a new tooth pushing through") and using a single question for the assessment of frequency of oral impacts*. A researcher was available to identify potential difficulties in completing the self-administered questionnaire and address queries by the children.

The self-administered questionnaire also contained socio-demographic information; age, sex and postcode. The postcode data was provided by the parent and was used to calculate the level of social deprivation using the Index of Multiple Deprivation (IMD) [26] that combines indicators across seven domains (income deprivation, employment deprivation, health deprivation and disability, education, skills and training deprivation, barriers to housing and services, living environment deprivation and crime) into a single deprivation rank. Based on the aforementioned characteristics, IMD scores are available for every postcode in England. According to the IMD distribution of the study sample, participants were categorised into high (two highest IMD quintiles) and low (three lowest IMD quintiles) deprivation groups. In addition, the self-administered questionnaire for the children contained questions about self-rated oral health status (5-point scale ranging from "very poor" to "very good"), self-perceived need for braces (4-point scale from "not at all" to "a lot"), frequency of thinking about dental appearance (5-point scale from "never" to "almost all the time") and satisfaction with dental appearance (5-point scale from "very unhappy" to "very happy"). Based on their distribution in the sample, they were all further categorised into 3-point scales for the analysis.

### Data analysis

The analysis started with a baseline comparison of socio-demographic characteristics and self-perceived measures between the SAQ and FTFI groups. This was done using Chi-square test or Mann-Whitney test. In addition, performance and overall Child-OIDP scores for both groups were calculated and statistically compared through the Mann-Whitney test.

Thereafter, the psychometric properties for the self-administered and interviewer-administered Child-OIDP questionnaire were first assessed individually, and then, compared with each other. In this study, the psychometric testing refers to criterion validity and internal reliability (consistency).

*Criterion validity *of each mode of administration was assessed against 4 proxy measures because of the lack of a gold standard to measure oral health-related quality of life. First, the correlation of the Child-OIDP score with four subjective oral health measures (self-rated oral health status, self-perceived need for braces, frequency of thinking about dental appearance and satisfaction with dental appearance) was estimated for each group by means of the Spearman's rho coefficient. Then, each one of these four correlations was compared between the FTFI and SAQ groups using Fisher's Z-transformation; that is, changing correlation values to Z-scores, and then using Fisher's Z test for the statistical comparison [27,28].

To assess the *internal reliability *of each mode of administration, inter-item correlations among the 8 performances scores were calculated as correlation matrices. Then, the comparison of the 28 inter-item correlations between the FTFI and SAQ groups was carried out in 2 stages: correlations were first compared as matrices using an asymptotic Chi-squared test [29], and if a difference was found at that level, individual comparisons were subsequently performed, using Fisher's transformation, to identify which ones of the 28 inter-item correlations differed between groups.

Finally, corrected item-total correlations and Cronbach's alpha coefficient were also calculated for each group. The item-total correlations were then compared between groups using the Fisher's Z transformation whereas Cronbach's alphas were compared by means of the Feldt's W test [30].

## Results

Baseline comparison between FTFI and SAQ groups is shown in Table 1. There was no statistically significant difference between the two groups in any of the socio-demographic characteristics (sex, age, social deprivation), as well as in the four self-perceived variables evaluated (p ≥ 0.614 and p ≥ 0.422 in all cases respectively). Similarly, there were no statistically significant differences when the different performances and overall scores were compared between the FTFI and SAQ groups (p ≥ 0.206 in all cases). And the same was the case for the comparison between the two groups in relation to the prevalence of the different performances and the overall Child-OIDP (p ≥ 0.165 in all cases). Furthermore, most performances had quite similar scores between the two groups. The mean overall Child-OIDP scores for the FTFI and SAQ groups were identical (3.16) (Table 2).

**Table 1:** Comparison of socio-demographic and self-perceived variables between face-to-face interview (FTFI) and self-administrated questionnaire (SAQ) groups

**Characteristics**	**FTFI group (n = 72)**		**SAQ group (n = 72)**		**p value**
		
	**n**	**%**	**n**	**%**	
Sex*					0.614
Boys	33	45.8	30	41.7	
Girls	39	54.2	42	58.3	
Social deprivation*					0.731
Low deprivation	26	36.1	28	38.9	
Higher deprivation	46	63.9	44	61.1	
*Age*					0.959
Mean ± S.D.	12.18 ± 1.59		12.24 ± 2.02		
*Self-rated oral health status*					0.508
Poor	4	5.6	7	9.8	
Fair	24	33.3	24	33.3	
Good	44	61.1	41	56.9	
*Self-perceived need to wear braces*					0.947
A little	19	26.4	17	23.6	
Maybe	23	31.9	26	36.1	
A lot	30	41.7	29	40.3	
*Happiness with dental appearance*					0.554
Unhappy	36	50.0	37	51.4	
No bothered	19	26.4	24	33.3	
Happy	17	23.6	11	15.3	
*Frequency of thinking of dental appearance*					0.422
Not often	17	23.6	13	18.1	
Sometimes	28	38.9	42	58.3	
A lot	27	37.5	17	23.6	

* Chi-square was used instead of the Mann-Whitney test

**Table 2:** Comparison of performances and overall scores between face-to-face interview (FTFI) and self-administrated questionnaire (SAQ) groups

**Performances**	**FTFI group (n = 72)**		**SAQ group (n = 72)**		**p value**
		
	**Mean**	**S.D**.	**Mean**	**S.D**.	
Eating	0.51	1.10	0.38	0.96	0.403
Speaking	0.40	1.35	0.29	0.97	0.507
Cleaning mouth	0.22	0.56	0.60	1.53	0.453
Sleeping	0.15	0.60	0.07	0.31	0.494
Emotion	0.11	0.36	0.10	0.34	0.775
Smiling	0.57	1.61	0.58	1.55	0.991
Studying	0.03	0.17	0.03	0.17	1.000
Social contact	0.28	0.74	0.24	0.86	0.206
*Overall impacts*	3.16	5.05	3.16	5.33	0.589

Mann-Whitney test was used

In relation to differences in the criterion validity testing between the two modes of administration, the Child-OIDP score was significantly correlated in the FTFI group to frequency of thinking about dental appearance, happiness with dental appearance, as well as self-perceived need for braces (p = 0.001, 0.015 and 0.027 respectively), but not to the self-rated oral health status (p = 0.747). On the other hand, in the SAQ group the Child-OIDP score was correlated significantly only to the question on happiness with dental appearance (p = 0.039), but not to the frequency of thinking about dental appearance, self-perceived need for braces or self-rated oral health status (p = 0.297, 0.325 and 0.346 respectively). Nevertheless, there was no statistically significant difference when each correlation coefficient was compared between the two modes of Child-OIDP administration. In addition, irrespective of their statistical significance, the direction of all examined associations followed the expected pattern, depending on the wording of the correlated variables, and it was similar for the FTFI and SAQ groups; namely, negative for self-rated oral health and happiness with dental appearance and positive for self-perceived need for braces and frequency of thinking about dental appearance (Table 3).

**Table 3:** Comparison of overall Child-OIDP scores between face-to-face interview and self-administered questionnaire groups

**Proxy measures**	**rho**	**p value**
*FACE-TO-FACE INTERVIEW GROUP (n = 72)*		
Self-rated oral health status (poor/.../good)	-0.04	0.747
Self-perceived need for braces (a little/.../a lot)	0.26	0.027
Happiness with dental appearance (unhappy/.../happy)	-0.28	0.015
Frequency of thinking about dental appearance (not often/.../a lot)	0.37	0.001
*SELF-ADMINISTERED QUESTIONNAIRE GROUP (n = 72)*		
Self-rated oral health status (poor/.../good)	-0.11	0.346
Self-perceived need for braces (a little/.../a lot)	0.12	0.325
Happiness with dental appearance (unhappy/.../happy)	-0.24	0.039
Frequency of thinking about dental appearance (not often/.../a lot)	0.13	0.297
*COMPARISON OF CORRELATIONS BETWEEN GROUPS*		
Self-rated oral health status (poor/.../good)	---	0.662
Self-perceived need for braces (a little/.../a lot)	---	0.394
Happiness with dental appearance (unhappy/.../happy)	---	0.787
Frequency of thinking about dental appearance (not often/.../a lot)	---	0.118

Spearman's rho correlation coefficient was used

(*) Fisher's Z-transformation was used

The inter-item correlations for the FTFI and SAQ groups were estimated as correlation matrices for the internal reliability analysis of each mode of administration. Two out of 28 inter-item correlations in the FTFI group and 8 out of 28 inter-item correlations in the SAQ group were negative. However, none of them was statistically different from zero (p > 0.05 in all cases). A statistically significant difference was found when the correlation matrices were compared between both groups using an asymptotic Chi-squared test (p < 0.001). But during the subsequent individual comparisons, only 6 out of 28 inter-item correlations differed between the FTFI and SAQ groups (p < 0.05), with 3 of these 6 correlations including the performance on social contact.

The corrected item-total correlations ranged between 0.13 and 0.52 for the FTFI group and between -0.04 and 0.48 for the SAQ group (Table 4). Cronbach's alpha coefficient was 0.54 and 0.55 for the FTFI and SAQ groups respectively. The alpha coefficient decreased when any performance was deleted from the Child-OIDP, with the exception of sleeping and speaking in the SAQ group. None of the item-total correlation coefficients was statistically different between the FTFI and SAQ groups (p > 0.086 in all cases). The same was the case for Cronbach's alpha (p = 0.466).

**Table 4:** Comparison of internal reliability between face-to-face interview (FTFI) and self-administrated questionnaire (SAQ) groups

**Performances**	**FTFI group (n = 72)**		**SAQ group (n = 72)**	
	
	**Item-total correlation^+^**	Alpha if item deleted*	**Item-total correlation^+^**	Alpha if item deleted*
Eating	0.24	0.52	0.44	0.46
Speaking	0.30	0.49	0.15	0.56
Cleaning mouth	0.16	0.53	0.36	0.49
Sleeping	0.19	0.53	-0.04	0.57
Emotion	0.42	0.50	0.15	0.55
Smiling	0.38	0.47	0.41	0.46
Studying	0.13	0.54	0.24	0.55
Social contact	0.52	0.43	0.48	0.45

Cronbach's alpha*	0.54		0.55	

^+ ^Fisher's Z-transformation was used

* Feldt's W test was used

## Discussion

This is the first study that assessed potential differences between the original interviewer-administered and the self-administered Child-OIDP. The self-administered version of the Child-OIDP performed similarly to the interviewer-administered original version of the instrument. Despite the demonstrated appropriateness of the interviewer-administered Child-OIDP in different cultural settings [1,25,31,32], an equivalent self-administered instrument would be more brief and cost-effective than face-to-face interviews, as it would not require an interviewer for administering the questionnaire. This would make the index more applicable in clinical settings, as it would be less disruptive to clinic routines. Furthermore, it would also favour its wider use in epidemiological studies of child populations, usually carried out in school settings where brevity and supervision have a considerable impact on resources needed.

This study shows the potential of the self-administered Child-OIDP to give comparable results to the interviewer-administered version, while at the same time reducing administrative burden. Children took less than 5 minutes to complete the self-administered Child-OIDP, without needing any help or clarification. This highlights its brevity and ease of understanding. Furthermore, unlike studies on self-administered questionnaires [11,33] and in contrast to a previous study using the OIDP which indicated lower response rates for a self-administered version [16], all children in this study fully completed the self-administered questionnaire, without requiring any clarifications or having queries about it, hence showing that the self-administration of the Child-OIDP was not associated with lower response rates or missing responses. This is encouraging and indicates that the concepts and wording of the Child-OIDP questions are appropriate for this age group, especially taking into consideration that a few children were quite young (9–10 year-olds), when comprehensiveness of complex questions goes beyond their capability and cognitive development [18].

In order to avoid any potential for bias in the comparison between the two modes, the sample was randomly divided into two equal groups and the comparison between the self-administered and the original interviewer-administered Child-OIDP was based on comparisons between the two groups. This approach has also been used in previous studies on modes of questionnaire administration for the assessment of sexual behaviour [8], smoking behaviour [9] and mental health [34]. In addition to their random selection, the comparability between the two groups was also established by showing no differences in their sociodemographic and subjective oral health backgrounds.

The similarities in the results between the self-administered and the original interviewer-administered Child-OIDP covered a variety of different aspects. First, there were no differences between the two groups in any of the eight performances or the overall Child-OIDP score. Moreover, most performances had similar mean scores for the two administration modes, while the overall Child-OIDP score was identical. More importantly, there were no differences between the self- and the interviewer-administered Child-OIDP in their associations with a number of different subjective measures of oral health that ranged from the broader measure of self-rated oral health status to questions more closely related to appearance and orthodontic treatment need, such as self-perceived need for braces. In terms of internal consistency, there was no significant difference between the FTFI and the SAQ groups in relation to any of the 8 item-total correlations as well as the overall alpha, while differences between the two groups were located in only 6 of the 28 examined inter-item correlations.

The results of this study are in accordance with previous studies assessing different questionnaire administration modes. They showed that there were no differences in health status and behaviours [7-10], and no differences in the psychometric performances related to health-related quality of life [12,13]. In line with a previous study using the OIDP [16] and one on EuroQoL [35], but in contrast to other relevant studies [6,12-15], we found no effect of the administration mode on the prevalence estimates of the outcome measure, as shown by the identical Child-OIDP scores between the two administration modes. However, the different settings and disease profiles of the samples limit comparability of our results with the aforementioned studies.

The nature of this study was to focus on differences between the two administration modes, not on the actual evaluation of the psychometric properties of the self-administered Child-OIDP. Indeed, the Cronbach's alpha was relatively low compared to respective figures from most other studies on Child-OIDP and some of the associations between the Child-OIDP and proxy measures were not significant. While the low value of alpha is affected by the nature of the Child-OIDP index and the fact that it contains only 8 items, the lack of statistical significance of the validity testing associations was mainly due to the limited sample size (n = 72 in each group), as we purposefully divided the sample into two groups in order to assess differences between the two modes of administration. Future studies with a different design, such as using the two administration modes per child to address the potential order effect and test-retest evaluation, are required to corroborate our results. The comprehensive psychometric evaluation of the self-administered Child-OIDP should be undertaken using a larger sample. Furthermore, this sample consisted of children referred for orthodontic treatment. A future study should also extend these results by using a general child population.

## Conclusion

The self-administered and the original interviewer-administered Child-OIDP performed similarly, thus providing support for the self-administration of the index. Further studies are needed on the comprehensive psychometric evaluation of the self-administered Child-OIDP.

## Declaration of competing interests

The authors declare that they have no competing interests.

## Authors' contributions

GT conceived of the study, participated in the study design and led the writing of the manuscript, EB carried out the data analysis and participated in writing the manuscript, KOB participated in the study design and critically reviewed the manuscript, AS conceived of the study and critically reviewed the manuscript, CO participated in the study design, carried out the data collection and participated in writing the manuscript. All authors read and approved the final manuscript.
